# Increased expression of immediate early response gene 3 protein promotes aggressive progression and predicts poor prognosis in human bladder cancer

**DOI:** 10.1186/s12894-018-0388-6

**Published:** 2018-09-24

**Authors:** Jianheng Ye, Yanqiong Zhang, Zhiduan Cai, Minyao Jiang, Bowei Li, Guo Chen, Yanru Zeng, Yuxiang Liang, Shulin Wu, Zongwei Wang, Huichan He, Weide Zhong, Chin-Lee Wu

**Affiliations:** 1 Department of Urology, Guangdong Key Laboratory of Clinical Molecular Medicine and Diagnostics, Guangzhou First People’s Hospital, Guangzhou Medical University, Guangzhou, 510180 China; 2 Departments of Urology and Pathology, Massachusetts General Hospital and Harvard Medical School, Boston, MA 02114 USA; 3 Institute of Chinese Materia Medica, China Academy of Chinese Medical Sciences, Beijing, 100700 China; 4 Southern Medical University, Guangzhou, 510515 China; 5 Department of Urology, Guangzhou First People’s Hospital, Guangzhou Medical University, Guangzhou, 510180 China; 6 Urology Key Laboratory of Guangdong Province, The First Affiliated Hospital of Guangzhou Medical University, Guangzhou Medical University, Guangzhou, 510230 China

**Keywords:** Immediate early response gene 3, Bladder cancer, Clinicopathological feature, Prognosis

## Abstract

**Background:**

Immediate early response gene 3 (IER3) is a stress-inducible gene, which exerts diverse effects in regulating cell apoptosis and cell cycle. Growing evidence shows that IER3 functions either as an oncogene or a tumor suppressor in various human cancers with a cancer type-dependent manner. However, the involvement of IER3 in human bladder cancer (BCa) has not been elucidated. In the current study, we aimed to investigate the expression pattern and the clinical significance of IER3 in BCa.

**Methods:**

We performed immunohistochemistry analysis to examine the subcellular localization and the expression levels of IER3 protein in 88 BCa specimens obtained from Department of Pathology in Massachusetts General Hospital. The associations of IER3 protein expression with various clinicopathological features and patients’ overall survival were statistically evaluated.

**Results:**

IER3 protein was mainly expressed in the cytoplasm in bladder cancer cell. Of 88 BCa tissue specimens, 39 (44.3%) showed high expression of IER3 protein and 49 (55.7%) showed low expression. High IER3 protein expression was significantly associated with high pathologic nodal stage (*p* = 0.018). Kaplan-Meier analysis revealed that the overall survival of BCa patients with overexpression of IER3 protein was shorter than that with low expression (*p* < 0.01). Multivariate analysis by Cox regression further identified IER3 as an independent prognostic factor of BCa patients (*p* = 0.010).

**Conclusions:**

Our findings suggest for the first time that the increased expression of IER3 protein may promote the aggressive progression of BCa. Importantly, IER3 may be a potential prognostic marker for BCa patients.

**Electronic supplementary material:**

The online version of this article (10.1186/s12894-018-0388-6) contains supplementary material, which is available to authorized users.

## Background

Bladder cancer (BCa) was the fourth most common cancer in men and the twelfth most common cancer in women in United States in 2016 [1]. Although there have been great advances in bladder carcinogenesis, the mortality rate of BCa is still high, due to its complex etiology and insufficient therapeutic strategies. A multivariate analysis showed that an increasing death rate of BCa was associated with multiple environmental exposures, such as smoking, air pollution, well water, urban residence and mining employment [2]. Most of BCa occur as non-muscle-invasive cancer, but there are still approximately 25% of them have muscle-invasive or metastatic disease, which leads to a poor outcome [3]. Therefore, it is of great clinical significance to discover novel and efficient molecular markers to develop more accurate diagnosis and prognosis methods for BCa patients.

Immediate early response gene 3 (IER3), also known as IEX-1, Dif-2, gly96 or p22/PRG-1, is a stress-inducible gene, which is rapidly regulated by multiple factors, including transcription factors, inflammatory cytokines, viral infection, chemical carcinogens, growth factors and hormones [4]. Under a wide range of stress, IER3 activation exerts diverse effects in regulating cell apoptosis and cell cycle with its distinct domains [5]. Accumulating studies have reported that IER3 may be associated with various signaling pathways, such as Nuclear factor kappa B (NF-κB) pathway and Mitogen-activated protein kinase (MAPK) /Extracellular regulated protein kinases (ERK) pathway, and may functions either as an oncogene or a tumor suppressor [6–8].

IER3 expression has been observed in a wide range of human epithelial tissues [9]. Growing evidence also shows that the aberrant expression of IER3 may be associated with prognosis in patients with multiple cancers [4]. However, the involvement of IER3 in BCa has not been elucidated. In this present study, we aimed to examine the expression pattern of IER3 protein in BCa tissues, and to evaluate its clinical significance in this malignancy.

## Methods

### Patients and tissues

This study used the same cohorts of BCa patients and tissue samples with our previous study [10]. All eighty-eight BCa tissue samples obtained from eighty-eight BCa patients who underwent cystectomy were collected from Massachusetts General Hospital between 2002 and 2010. The BCa patients’ detail clinical information was shown in Table 1. All cases were re-reviewed by CLW and JHY (two authors) according to the newest version of World Health Organization classification of tumor of the bladder [11].

**Table 1:** Associations between IER3 protein expression and various clinicopathological characteristics of 88 BCa patients underwent cystectomy between 2002 and 2010

	IER3 (low)	IER3 (high)	*P*
Number of patients, no. %	49(55.7)	39(44.3)	
Gender, no.%			0.799
Female	12(24.5)	8(20.5)	
Male	37(75.5)	31(79.5)	
Age at Surgery, median(IQR)	70(62–75)	72(62–80)	0.462
pT, no. %			0.493
< =pT2	16(32.7)	10(25.6)	
> =pT3	33(67.3)	29(74.4)	
pN, no. % (*n* = 79)			**0.018**
pN(−)	33(76.7)	18(50.0)	
pN(+)	10(23.3)	18(50.0)	
LVI, no. %			0.284
LVI(−)	31(63.3)	20(51.3)	
LVI(+)	18(36.7)	19(48.7)	
PNI, no. %			0.808
PNI(−)	37(75.5)	28(71.8)	
PNI(+)	12(24.5)	11(28.2)	
STSM, no. %			0.781
margin(−)	40(81.6)	33(84.6)	
margin(+)	9(18.4)	6(15.4)	
Metastasis, no. %			1.000
Mets(−)	29(59.2)	23(59.0)	
Mets(+)	20(40.8)	16(41.0)	

Bold values indicate that they are less than 0.05

### Immunohistochemistry and Immunoreactive score

Following the immunohistochemistry (IHC) protocol we described in our previous study [10], IER3 protein expression level was assessed by IHC using a polyclonal goat anti-IER3 antibody (Santa-Cruz biotechnology, CA). Detail information of IHC protocol was provided in Additional file 1. After performing IHC, the IER3 protein expression level in each tissue section was evaluated according to the immunostaining intensity and percentage. Immunoreactive score (IRS) was obtained by multiplying intensity and percentage of immunostaining. The intensity of immunostaining graded from 0 to 3: 0 (negative), 1 (weak), 2 (moderate), and 3 (strong); the percentage of staining cells were classified as 0–4: 0 (negative), 1 (<=10%), 2 (11–50%), 3 (51–80%), and 4 (> 80%). The final IRS (ranged from 0 to 12) was determined: 0–3 as low IER3 protein expression, and 4–12 as high IER3 protein expression [12]. The IRS of IER3 immunostaining of each tissue section was scored by two investigators (CLW and JHY) independently in a blind manner without any knowledge of the patient clinical characteristics. The IRSs evaluated by two investigators respectively were compared. In order to reach a consensus, different IRSs of the same tissue section were reevaluated through a re-examination of the intensity of IER3 immunostaining by both investigators.

### Statistics

All statistical analyses in this study were performed using Stata14 software (College Station, TX, USA).The associations between expression level of IER3 protein and BCa patients’ clinicopathological data were analyzed by two statistical methods (Pearson’s Chi-squared test or Fisher’s exact test). Kaplan-Meier method was using to estimate the BCa patients’ overall survival. Then, log-rank test was used for assessing differences. Cox proportional hazard regression models were used to perform univariate and multivariate survival analyses. Hazard ratios (HR) and the corresponding 95% confidence intervals (CI) were used to express the relative risks of dying. Two-sided with *p* < 0.05 was considered as statistically significant.

## Results

### Protein expression of IER3 in BCa

**Fig. 1:**
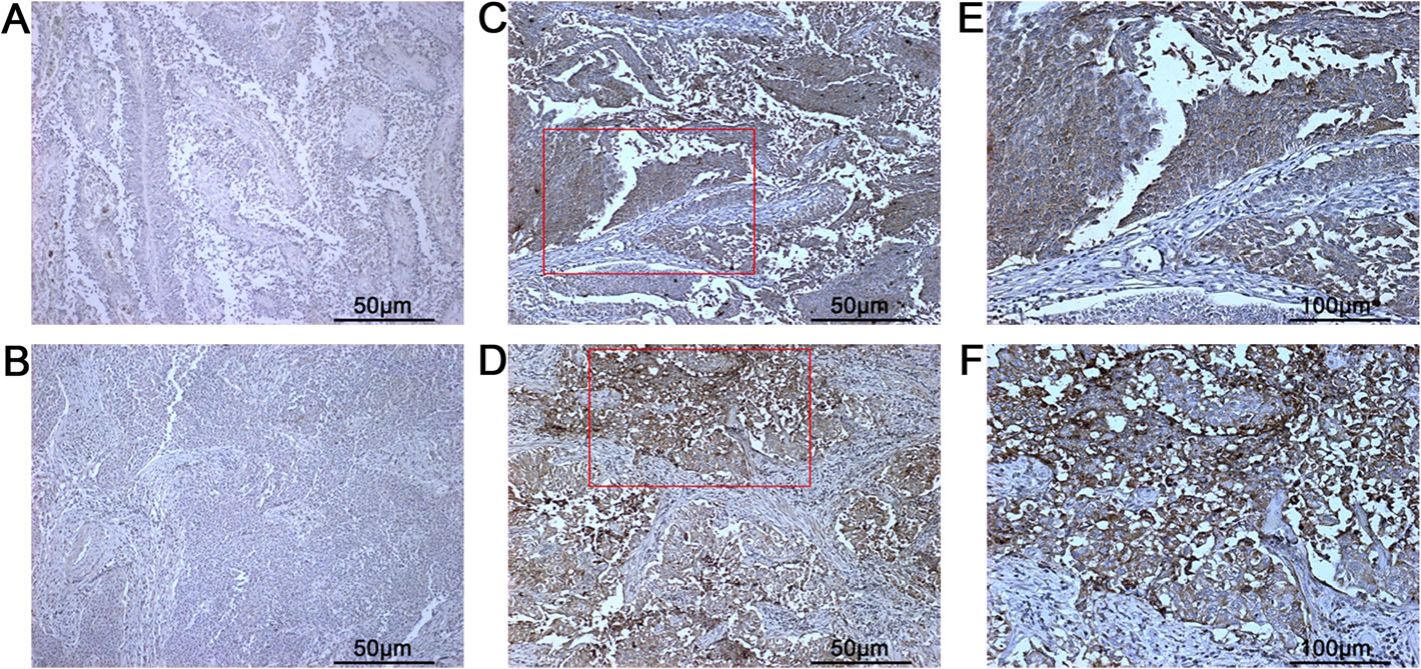
Representative images of IER3 immunostaining in BCa tissue specimens. Negative (**a** and **b**) and positive (**c** and **d**) expression of IER3 protein are shown (Magnification: 100X). Magnified images (**e** and **f**) of two immunostaining regions marked by red boxes in **c** and **d** (Magnification: 200X)

IER3 protein’s expression in 88 BCa patients’ tissue samples was identified by IHC. As shown in Fig. 1, IER3 protein was mainly localized in the cytoplasm of BCa cells. All specimens were divided into 2 groups: high IER3 protein expression group, containing 39 (44.3%) BCa patients; low IER3 protein expression group, containing 49 (55.7%) BCa patients. Statistical analysis showed that BCa patients with high expression of IER3 protein more frequently had high pathologic nodal stage (pN) (*p* = 0.018, Table 1). However, no significant associations were observed between IER3 protein expression level and other clinicopathological features, including patients’ age, pathological stage (pT), gender, perineural invasion (PNI), lymphvascular invasion (LVI), soft tissue surgical margins (STSM) and distant metastasis.

### High IER3 protein expression was an independent indicator of poor prognosis in BCa patients

**Fig. 2:**
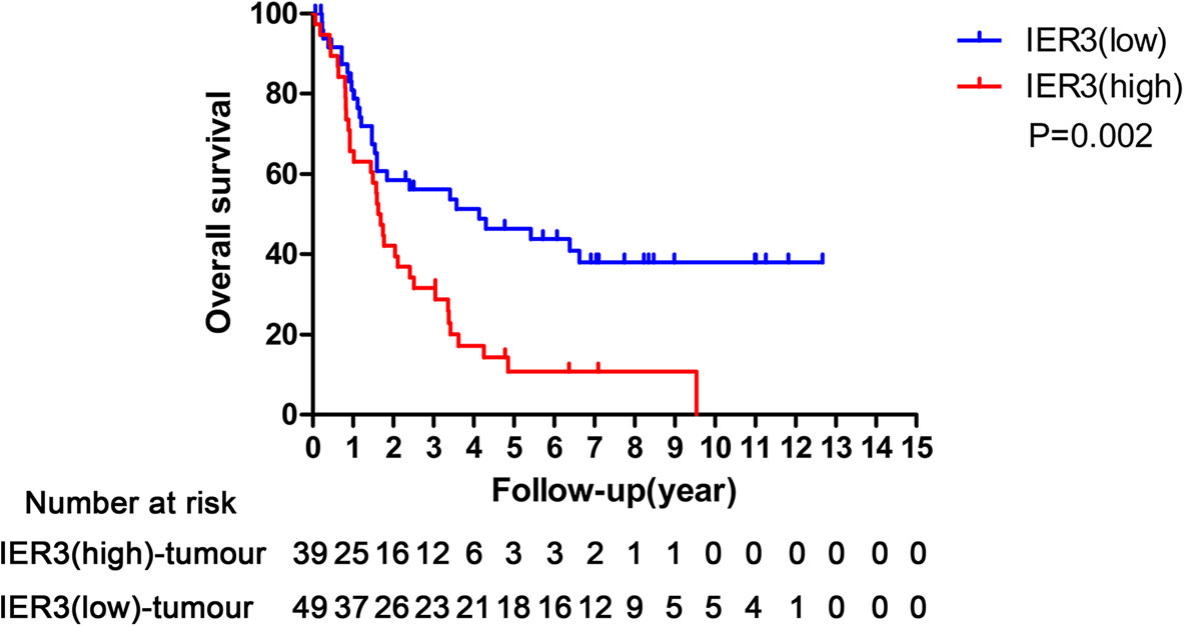
Kaplan-Meier curves representing the overall survival of 88 patients treated with cystectomy for bladder cancer stratified by IER3 status (*p* = 0.002)

After analyzed by Kaplan-Meier method, the curves showed that BCa patients in high IER3 protein expression group had worse overall survival compared with those in low IER3 protein expression group (*p* = 0.002,Fig. 2). Additionally, the univariate analysis demonstrated that old patients’ age (*p* = 0.029), high pT (*p* = 0.006), high pN (*p* < 0.001), positive LVI (*p* = 0.006), positive STSM (*p* = 0.002), positive distant metastasis (*p* = 0.001) and high expression of IER3 protein (*p* = 0.003) were all associated with poor survival of BCa patients significantly (Table 2). The multivariate survival comparison indicated that patients’ age (*p* = 0.012), pN (*p* = 0.007) and IER3 protein expression (*p* = 0.010) can act as the independent prognostic indicators of BCa (Table 2).

**Table 2:** Prognostic value of IER3 protein expression for the overall survival by Cox proportional hazards model

	Univariable			Multivariable		
	Hazard Ratio	95% CI	*P*	Hazard Ratio Ratio	95% CI	*p*
Gender						
Male vs. Female	1.14	0.62–2.10	0.681			
Age	1.03	1.00–1.05	**0.029**	1.04	1.01–1.07	**0.012**
pT						
> =pT3 vs. <=pT2	2.39	1.29–4.42	**0.006**	1.54	0.71–3.32	0.272
pN						
pN(+) vs. pN(−)	3.41	1.95–5.99	**< 0.001**	2.35	1.26–4.38	**0.007**
LVI						
LVI(+) vs. LVI(−)	2.03	1.22–3.37	**0.006**	0.99	0.52–1.89	0.987
PNI						
PNI(+) vs. PNI(−)	1.03	0.56–1.87	0.935			
STSM						
STSM(+) vs. STSM(−)	2.62	1.41–4.88	**0.002**	2.02	0.96–4.27	0.065
Metastasis						
Mets(+) vs. Mets(−)	2.35	1.41–3.97	**0.001**	1.81	0.98–3.33	0.058
IER3 IHC						
IER3(high) vs. IER3(low)	2.16	1.29–3.13	**0.003**	2.21	1.21–4.04	**0.010**

Bold values indicate that they are less than 0.05

## Discussion

IER3 was initially identified as a radiation-inducible protein in squamous carcinomas [13]. It has been demonstrated to response to quite distinct stress or cellular stimuli immediately. Among these stimulating factors, NF-κB, p53, SP1 and AP1 are well-known to be involved into tumor development [6]. Increasing evidence show that IER3 may play complex roles in cell apoptosis in different manners [4–6]. In a mitochondria-dependent environment, IER3 up-regulated can reduce the production of intracellular reactive oxygen species by facilitating the degradation of the inhibitor of F1 catalytic sector, which can prevent apoptosis at its initial phase [14]. On the other hand, IER3 was identified to mediate the NF-κB signaling pathway by interacting with RelA/p65 subunit. This modification can contribute to inducing the expression of anti-apoptotic NF-κB target genes and then supporting cell apoptosis [15]. Besides the impact of IER3 on NF-κB signaling pathway, some studies indicated the IER3 might act as a regulator of ERK1/2 pathway. A phenomenon found by Letourneux et al. [16] showed that ERK activation led to IER3 phosphorylation. Simultaneously, the p-IER3 can enhance ERK phosphorylation by preventing its dephosphorylation factor B56-containing PP2A. The roles of IER3 sustaining ERK1/2 phosphorylation and promoting the tumor development were found in pancreatic cancer [17], lung adenocarcinoma [18] and Hodgkin lymphoma [19]. Moreover, IER3 were found to play crucial roles in regulating tumor growth. Han et al. [20] analyzed the clinical significance of IER3 expression in 77 ovarian carcinoma patients by using immunohistochemistry, and found a decreased expression of IER3 in ovarian carcinoma tissues compared to cystadenoma and borderline tumors tissues. They also indicated that the decreased expression of IER3 was associated with a short survival time of patients with this cancer. They subsequently showed a positive correlation between IER3 expression and anti-apoptotic activity of ovarian cancer cell. In breast cancer, estrogen was reported to effectively up-regulated the expression of IER3 [21]. Furthermore, Yang et al. [22] showed IER3 was overexpressed in invasive breast cancer tissues compared with preinvasive cancer tissues. In vitro experimental system, IER3 was found to reduce the apoptotic activity of T47D and MCF-7 cells. Akilov et al. [23] revealed that IER3 up-regulation in Sézary cells might result in decreased expression levels of reactive oxygen species; as a downstream target of TNF-α-induced pathway, IER3 could protect Sézary cells from TNF-α-induced apoptosis. Sasada et al. [24] found that IER3 protein showed high expression in 41 patients and low expression in 37 patients with pancreatic cancer; statistically, patients with IER3 overexpression had a significantly better survival than those with low expression. In colon cancer, Nambiar et al. [25] built two different mouse models with hyperplasic or dysplastic preneoplastic aberrant crypt foci (ACF). The gene expression analysis based on ACF lesions showed that IER3 expression was increased in low risk ACF mice compared to high risk ACF mice; further immunohistochemistry analysis showed the positive staining of IER3 in adjacent normal-appearing colonic while the negative staining in the tumor crypts from the same patients. These findings suggest that the aberrant expression of IER3 may be implicated into carcinogenesis, progression and patients’ outcome of various human cancers.

In present study, we determined that IER3 protein was overexpressed in the cytoplasm of cancer cells in BCa tissues specimens and that high IER3 protein expression was distinctly associated with high pathologic nodal stage. Notably, high IER3 protein expression also predicted poor overall survival in BCa patients.

## Conclusions

Our findings suggest for the first time that the increased expression of IER3 protein may promote the aggressive progression of BCa. Importantly, IER3 may be a potential prognostic marker for BCa patients. Further investigations on molecular mechanisms underlying IER3 involvement in BCa are required.

## Additional file

Additional file 1: Immunohistochemistry Protocol. (DOCX 23 kb)

## Abbreviations

ACF: Aberrant crypt foci
AP-1: Activator protein 1
BCa: Bladder cancer
CI: Confidence intervals
ERK: Extracellular regulated protein kinases
HR: Hazard ratios
IER3: Immediate early response gene 3
IHC: Immunohistochemistry
IQR: Interquartile Range
IRS: Immunoreactive score
LVI: Lymphvascular invasion
MAPK: Mitogen-activated protein kinase
Mets: Metastasis
NF-κB: Nuclear factor kappa B
P: *p-*value
pN: Pathologic nodal stage
PNI: Perineural invasion
pT: Pathological stage
SP1: Specificity protein 1
STSM: Soft tissue surgical margins
TNFα: Tumor necrosis factor alpha
