# Supplementary material for: Increased expression of immediate early response gene 3 protein promotes aggressive progression and predicts poor prognosis in human bladder cancer
Source: BMC Urol. 2018 Sep 24;18:82. doi: 10.1186/s12894-018-0388-6 (PMC6154418; doi:10.1186/s12894-018-0388-6)
Supplement: Supplementary file 1 — Immunohistochemistry Protocol. (DOCX 23 kb) [file 12894_2018_388_MOESM1_ESM.docx]

Immunohistochemistry Protocol

The BCa tissue samples were fixed in 10% neutral-buffered formalin and embedded in paraffin. Place slides in holder and dry in 60℃ over for 30 mins (up to 45 mins) to remove paraffin. After the slides deparaffinized in xylene and rehydrated in a graded ethanol series, antigen retrieval was performed in R-buffer A (Electron Microscopy Science) using a pressure cooker. Then, the endogenous peroxidase activity was blocked with 3% H_2_O_2_. Nonspecific binding was blocked with 5% horse serum in TBST for 30 minutes. Slides were then incubated overnight with the primary anti-IER3 antibody (Santa-Cruz biotechnology, CA) at a dilution of 1: 100, at 4℃. After washing three times in TBST, slides incubated with anti-goat secondary antibody (Vector Labs, Burlingame, CA) at a dilution of 1: 300. After washing three times in TBST, slides were placed in ABC solution (Vector Labs, Burlingame, CA) for 30 mins on shaker. The peroxidase reaction was developed using DakoCytomation Liquid DAB plus Substrate Chromogen System (DakoCytomation).
